# The Role of Natural Language Processing in Graduate Medical Education: A Scoping Review

**DOI:** 10.7759/cureus.81078

**Published:** 2025-03-24

**Authors:** Ravi K Janumpally

**Affiliations:** 1 Clinical Informatics, Baylor Scott & White Medical Center, Round Rock, USA

**Keywords:** graduate medical education, large language models, natural language processing, natural language processing (nlp), residency training

## Abstract

The rapid evolution of artificial intelligence, particularly in the form of natural language processing (NLP) and large language models (LLMs), presents new opportunities to enhance graduate medical education (GME). NLP technologies have the potential to improve residency training programs by automating performance feedback, personalizing learning pathways, and identifying competency gaps. However, the integration of these technologies also raises challenges related to privacy, ethical considerations, and algorithmic bias. This review provides a comprehensive evaluation of the application and impact of NLP in GME.

A scoping review of the literature was conducted following the Preferred Reporting Items for Systematic Reviews and Meta-Analyses (PRISMA) guidelines. Relevant studies from 2018 to 2024 were identified using databases such as PubMed, Scopus, Web of Science, and Google Scholar. Inclusion criteria focused on peer-reviewed studies evaluating NLP applications in residency training programs across various specialties. Data were extracted from 20 studies, and key themes were synthesized to assess the educational, technological, and ethical implications of NLP in GME.

The review identified several key areas where NLP is transforming GME. These include automated performance evaluation systems, sentiment analysis of narrative feedback, personalized learning recommendations, and competency assessment algorithms. NLP technologies demonstrated significant potential in reducing administrative workload, improving assessment accuracy, and enhancing the personalization of residency training. However, studies also highlighted concerns regarding algorithmic biases and the need for transparent, ethical frameworks to ensure fair implementation.

The integration of NLP in GME offers significant opportunities to streamline educational processes and enhance trainee development. Automated feedback systems can reduce subjective biases and provide more actionable insights for residents. Additionally, NLP applications can identify early indicators of residents at risk of underperformance and support timely interventions. However, the adoption of these technologies requires careful consideration of ethical and legal implications, particularly around data privacy and fairness.

NLP has the potential to revolutionize GME by improving the quality and efficiency of residency training programs. While the technology offers promising benefits, further research is needed to address ethical challenges and ensure responsible implementation. Interdisciplinary collaboration between educators, informaticians, and ethicists will be critical to fully realize the potential of NLP in medical education.

## Introduction and background

Natural language processing (NLP) is a subset of machine learning (ML) focused on enabling computers to understand, interpret, and generate human language, often relying on statistical and deep learning techniques. Generative artificial intelligence (AI), a broader category that includes models like the generative pre-trained transformer (GPT), leverages ML, including NLP, to create new text, images, or other content, distinguishing it from traditional ML approaches that primarily focus on pattern recognition and prediction rather than content generation.

The rapid advancement of AI and large language models (LLMs) has precipitated a transformative wave across numerous professional domains, with graduate medical education (GME) emerging as a particularly promising frontier for technological innovation [1].NLP technologies have demonstrated remarkable potential to revolutionize multiple aspects of residency training programs, encompassing educational strategies, clinical training methodologies, and resident performance assessment [2,3].

GME faces significant challenges in standardizing evaluation, personalizing learning experiences, and efficiently managing the vast amounts of textual data generated during training [4]. Traditional assessment methods often rely on subjective narratives, handwritten evaluations, and time-consuming feedback mechanisms that are prone to human bias, inconsistency, and sometimes gender bias [5,6]. NLP offers unprecedented opportunities to address these systemic limitations by providing sophisticated, data-driven approaches to analyzing and interpreting complex educational and clinical interactions [7].

The application of NLP in residency training programs spans several critical domains. These include automated performance feedback analysis, competency assessment, and the extraction of meaningful insights from resident-generated documentation [8,9]. ML algorithms can now deconstruct narrative evaluations, identify nuanced performance patterns, and generate actionable recommendations that support both individual resident development and programmatic improvement [10].

Recent studies have highlighted the potential of NLP to enhance several key educational processes. For instance, sentiment analysis of evaluation narratives can provide a more nuanced understanding of resident performance beyond traditional grading systems [11,12]. Automated text classification techniques enable more efficient categorization of clinical competencies, reducing administrative burden and improving assessment accuracy [13]. Moreover, natural language understanding technologies can help identify early indicators of resident struggle or exceptional performance, allowing for more proactive educational interventions [14].

Internationally, the adoption of NLP in GME reveals diverse implementation strategies. While United States residency programs have been at the forefront of technological integration, countries like Canada and China have also begun exploring sophisticated NLP applications in medical training [15-17]. These global perspectives underscore the universal potential of AI-driven educational technologies to transform medical professional development.

However, the implementation of NLP in GME is not without challenges. Significant considerations include maintaining patient privacy, ensuring algorithmic fairness, addressing potential biases in training data, and navigating complex ethical landscapes [18]. The responsible development and deployment of these technologies require interdisciplinary collaboration among medical educators, computational experts, and ethicists [19,20].

As LLMs continue to evolve, their potential to revolutionize GME becomes increasingly apparent. The ability to process, understand, and generate human-like text offers unprecedented opportunities for personalized learning, comprehensive assessment, and continuous improvement of medical training programs [21-24]. Research must focus on developing robust, ethically sound NLP frameworks that can be seamlessly integrated into existing educational infrastructures [25,26]. Thus, this review aims to provide a comprehensive, nuanced understanding of NLP's transformative potential in GME, balancing technological innovation with pedagogical effectiveness and ethical considerations.

## Review

Research aims, objectives, and framework

Overall Research Question

The research investigated the current landscape, effectiveness, and potential impact of NLP technologies in GME, specifically within residency training programs, across educational, clinical training, and assessment domains.

Specific Objectives

This study aimed to comprehensively explore the role of NLP technologies in residency training by addressing several dimensions. It sought to conduct technological mapping, identifying and categorizing the various NLP technologies utilized in medical education. This included analyzing their technological sophistication and computational approaches to better understand how they supported residency training programs. The study also focused on evaluating the educational impact of NLP tools in improving critical aspects of medical education, including their role in resident performance assessment, the development of personalized learning pathways, enhancements in curriculum design, and the refinement of feedback mechanisms. A comparative analysis was performed to investigate NLP implementation strategies across different medical specialties and geographic regions. This analysis identified variations in technological adoption and educational outcomes, providing a global perspective on the integration of NLP into medical education. Additionally, the study addressed ethical considerations and implementation challenges by examining barriers to adoption and proposing solutions to the technological and pedagogical challenges that arose during integration. It also explored the future trajectory of NLP in GME by predicting emerging trends in its application and recommending evidence-based strategies to ensure successful technological integration in the years to come. Through these efforts, the project aspired to provide a comprehensive framework for understanding and advancing NLP technologies in medical education. The overall research question and specific objectives of the study are outlined in the PICO (population, intervention, comparison, and outcomes) framework in Table 1.

**Table 1:** PICO framework. PICO: population, intervention, comparison, and outcomes.

PICO component	Detailed description
P (Population)	- Medical residents in graduate training programs
	- Specific specialties: Internal medicine, surgery, pediatrics, psychiatry, emergency medicine
	- Geographic focus: United States, Canada, United Kingdom, Australia, European Union
	- Exclusion: Undergraduate medical students, fellows, practicing physicians
I (Intervention)	- Natural language processing (NLP) technologies
	- Large language models (LLMs)
	- Artificial intelligence-driven educational tools
	- Specific applications:
	* Automated performance evaluation systems
	* Intelligent feedback generation
	* Personalized learning path recommendations
	* Clinical documentation analysis
	* Competency assessment algorithms
C (Comparison)	- Traditional medical education assessment methods
	- Manual evaluation processes
	- Conventional feedback mechanisms
	- Human-only performance assessment
	- Pre-NLP educational technologies
O (Outcomes)	Primary outcomes:
	- Accuracy of resident performance assessment
	- Quality and specificity of educational feedback
	- Personalization of learning experiences
	- Reduction in administrative workload
	- Improvement in resident skill development
	Secondary outcomes:
	- Technological adoption rates
	- Cost-effectiveness of NLP implementations
	- User satisfaction (residents and educators)
	- Ethical considerations and potential biases
	- Long-term educational and clinical performance impacts

Methodology

**Figure 1:**
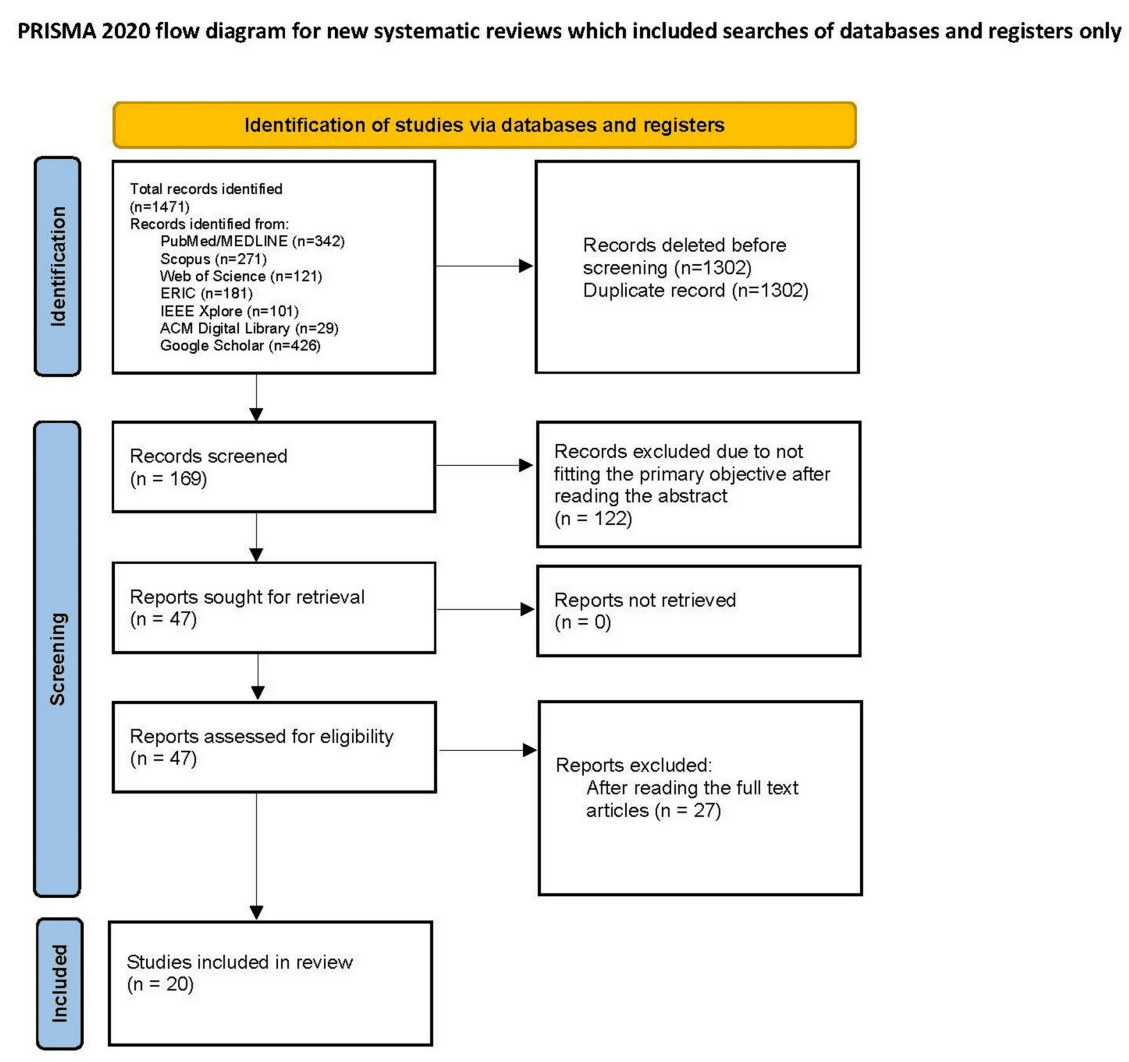
PRISMA diagram. PRISMA: Preferred Reporting Items for Systematic Reviews and Meta-Analyses.

The scoping review was conducted and reported in accordance with the Preferred Reporting Items for Systematic Reviews and Meta-Analyses Extension for Scoping Reviews (PRISMA-ScR) 2018 guidelines [27]. The Preferred Reporting Items for Systematic Reviews and Meta-Analyses (PRISMA) diagram is presented in Figure 1. The study employed rigorous eligibility criteria to ensure the inclusion of relevant and high-quality research on the application of NLP and LLMs in GME. The target population included residents enrolled in GME programs worldwide, with a primary focus on residency training programs in the United States, Canada, and the United Kingdom. Studies involving undergraduate medical education were excluded from the scope. The interventions of interest included studies that applied NLP and LLM technologies to resident education, clinical training, performance assessment, curriculum development, and feedback mechanisms. Eligible study types comprised peer-reviewed original research, including retrospective and prospective studies, randomized controlled trials, observational studies, and quasi-experimental studies. Only studies published in English between January 2018 and October 2024 and appearing in peer-reviewed journals were included.

A comprehensive search strategy was employed using several electronic databases, including PubMed/MEDLINE, Scopus, Web of Science, ERIC, IEEE Xplore, ACM Digital Library, and Google Scholar. The search strategy combined Boolean operators and Medical Subject Heading (MeSH) terms to identify studies relevant to NLP and LLM technologies in residency training. The search string in PubMed included the terms “Natural Language Processing” OR “Large Language Models” AND combined with “Graduate Medical Education“ OR "Residency” OR “Assessment” OR “Performance”. The study selection process followed a structured workflow. An initial database search identified potential studies, followed by duplicate removal. Screening occurred in two stages: title and abstract screening and a full-text review. One reviewer, the author RJ, conducted all stages of the review and extraction for this study. Unfortunately, multiple reviewers were not available for both screening stages. Full-text articles of potentially relevant studies were retrieved based on predefined criteria. Reasons for exclusion were documented.

Data extraction used a standardized and piloted form to capture key information from selected studies, including study identification details, study design, population characteristics, NLP methodology, specific technologies or models used, educational domains, key findings, limitations, and ethical considerations. The analysis involved qualitative synthesis, which employed thematic analysis to identify recurring patterns, context-specific insights, and trends in technological innovation. Together, these analyses provided a comprehensive understanding of the role and impact of NLP and LLM technologies in GME.

Results

Table 2 summarizes the key information from the 20 included studies [16,28-46].

**Table 2:** Summary of included studies pertaining to the role of NLP and LLM in graduate medical education. NLP: natural language processing; LLM: large language model; USMLE: United States Medical Licensing Examination; AOA: alpha-omega-alpha; ACGME: Accreditation Council for Graduate Medical Education; AUROC: area under the receiver operating characteristic curve; AUPRC: area under the precision-recall curve; BERT: Bidirectional Encoder Representations from Transformers; TF-IDF: term frequency-inverse document frequency.

Sr. No.	Author(s)/year	Type of study	Study material	Purpose of study	Outcomes of study	Findings of study	Sample size (N)
1	Nori et al. (2023) [16]	Model evaluation study	Capabilities of GPT-4 on medical challenge problems	Evaluation of GPT-4's performance on USMLE Step 1–3 and other medical benchmarks	GPT-4 exceeded USMLE Step 3 passing score by over 20 points; improved calibration and reasoning vs. GPT-3.5	Demonstrates strong potential for GPT-4 as a tool in graduate medical education, especially for Step 3 preparation and AI-assisted learning	For Step 3, n = 267
2	Yilmaz et al. (2022) [28]	Retrospective	Workplace-based assessment (WBA) narrative comments	To explore NLP and machine learning (ML) applications for identifying trainees at risk using a large WBA narrative comment data set associated with numerical ratings.	NLP was performed on a data set of narrative comments derived from WBAs, and supervised ML models using linear regression were trained with the quantitative ratings to output a prediction of whether a resident fell into the category of at-risk or not at risk.	The ML models can accurately identify underperforming residents via narrative comments provided for WBAs, and the words generated in WBAs can be a worthy data set to augment human decisions for educators tasked with processing large volumes of narrative assessments.	N = 7199
3	Spadafore et al. (2024) [29]	Retrospective	2,500 entrustable professional activity (EPA) assessments from emergency medicine residency programs	To develop an NLP model for applying the Quality of Assessment for Learning (QuAL) score to narrative supervisor comments.	The NLP model trained on QuAL-rated EPA comments to predict overall and subcomponent scores, with performance assessed across improvement suggestions and performance link dimensions.	The model predicted the exact or near-exact QuAL score (±1) in 87% of cases, performing well in evaluating feedback quality, improvement suggestions, and the link between comments and resident performance.	N = 2,500
4	Solano et al. (2021) [30]	Retrospective	Surgical resident feedback transcripts	To validate the performance of a natural language processing (NLP) model in characterizing the quality of feedback provided to surgical trainees.	The NLP model classified the quality (high vs. low) of 2,416 narrative feedback transcripts with an accuracy of 0.83, sensitivity of 0.37, specificity of 0.97, and an area under the receiver operating characteristic curve of 0.86.	The NLP model classified the quality of operative performance feedback with high accuracy and specificity, offering residency programs the opportunity to efficiently measure feedback quality.	N = 2416
5	Sarraf et al. (2021) [31]	Retrospective	Letters of recommendation (LoRs) for general surgery residency candidates	To examine gender bias in LoRs written for surgical residency candidates across three decades at one institution.	LoRs were analyzed using artificial intelligence (AI) and natural language processing (NLP) to conduct sentiment analysis and detect gender bias.	AI and computer-based algorithms detected linguistic differences and gender bias in LoRs, even following stratification by clerkship grades and when analyzed by decade.	N = 611
6	Gudgel et al. (2021) [32]	Retrospective	Ophthalmology residency applications	To identify application attributes correlated with successful ophthalmology residency performance.	Residents were subjectively ranked into tertiles and top and bottom deciles based on residency performance, and various application attributes were analyzed for associations.	Many metrics traditionally felt to be predictive of residency success (USMLE scores, AOA status, and research) did not predict resident success. Core clerkship grades and medical school ranking were associated with high subjective ranking.	N = 76
7	Ötleş et al. (2021) [33]	Retrospective	Surgical trainee feedback comments	To evaluate whether NLP can be used to automatically classify the quality of surgical trainee evaluations.	NLP models were trained to automatically classify the quality of feedback across 4 categories (effective, mediocre, ineffective, or other).	The NLP model using a support vector machine algorithm yielded a maximum mean accuracy of 0.64 in classifying feedback quality. When the classification task was modified to distinguish only high-quality vs. low-quality feedback, the maximum mean accuracy was 0.83.	N = 600
8	Abbott et al. (2021) [34]	Retrospective	End-of-rotation assessments and clinical competency committee (CCC) assessments for surgical residents	To examine whether NLP can be used to estimate CCC ratings.	Models of end-of-rotation assessment ratings and text were created to predict dichotomized CCC assessment ratings for 16 ACGME milestones, with and without predictors derived from NLP of end-of-rotation assessment text.	NLP can identify language correlated with specific ACGME milestone ratings, and faculty could use information automatically extracted from text to focus attention on residents who might benefit from additional support and guide the development of educational interventions.	N = 691
9	Andrews et al. (2021) [35]	Retrospective	Internal medicine resident evaluations	To examine differences in word use, competency themes, and length within written evaluations of internal medicine residents, considering the impact of both faculty and resident gender.	Researchers developed a sentiment model to assess the valence of evaluation responses and used NLP to evaluate whether female versus male residents received more positive or negative feedback and if that feedback focused on different ACGME core competencies.	When examined at scale, quantitative gender differences were not as prevalent as has been seen in qualitative work, suggesting that further investigation of linguistic phenomena (such as context) is warranted to reconcile this finding with prior work.	N = 3864
10	Stahl et al. (2021) [36]	Retrospective	Entrustable professional activity (EPA) micro-assessments	To analyze actual EPA assessment narrative comments using NLP to enhance our understanding of resident entrustment in actual practice.	Latent Dirichlet allocation (LDA), a machine learning algorithm, was used to identify latent topics in the documents associated with a single EPA, which were then reviewed for interpretability by human raters.	LDA is capable of identifying topics relevant to progressive surgical entrustment and autonomy in EPA comments, providing insight into key behaviors that drive different levels of resident autonomy and may allow for data-driven revision of EPA entrustment maps.	N = 1015
11	Geller et al. (2022) [37]	Retrospective	Orthopedic surgery residency program social media presence	To analyze the changes in social media usage by orthopedic surgery programs in response to the COVID-19 pandemic.	Social media data (Instagram and Twitter) were collected and analyzed, including the total number of followers, accounts following, tweets, likes, date of account creation, hashtags, and mentions. Natural language processing was utilized for tweet sentiment analysis.	The study demonstrated substantial growth of Instagram and Twitter presence by orthopedic surgery residency programs during the COVID-19 pandemic, suggesting that programs have utilized social media as a new way to communicate with applicants and showcase their programs.	N = 85
12	Ortiz et al. (2023) [38]	Retrospective	Neurosurgery residency applications	To compare the performance of machine learning models trained on applicant narrative letters of recommendation (NLORs) and demographic data to predict match outcomes and investigate whether narrative language is predictive of standardized letter of recommendation (SLOR) rankings.	Logistic regression models using the least absolute shrinkage and selection operator were trained to predict match outcomes using applicant NLOR text and demographics, and another model was trained on NLOR text to predict SLOR rankings.	Both the NLOR and demographics models were able to discriminate similarly between match outcomes. Words including "outstanding," "seamlessly," and "AOA" were predictive of match success, and words like "highest," "outstanding," and "best" were predictive of the top 5% SLORs.	N = 1498
13	Drum et al. (2023) [39]	Retrospective	Internal medicine-pediatrics residency applications	To develop a machine learning model (MLM) that can identify several values important for resident success in internal medicine-pediatrics programs.	Expert reviewers annotated text snippets from the narrative sections of applications into specific values (e.g., academic strength, compassion, communication) associated with resident success. The authors then applied the MLM to prospective applications and compared the output with a manual holistic review.	The MLM had a sensitivity of 0.64 and a specificity of 0.97, and the mean total number of annotations per application was significantly correlated with invited for interview status.	N = 11,333
14	Mahtani et al. (2023) [40]	Retrospective	Residency application experience entries	To develop an NLP-based tool to automate the review of applicants' narrative experience entries and predict interview invitations.	Experience entries were extracted from residency applications, combined at the applicant level, and paired with the interview invitation decision. NLP identified important words (or word pairs), which were used to predict interview invitation using logistic regression.	The NLP model had an AUROC of 0.80 and AUPRC of 0.49, showing moderate predictive strength. Phrases indicating active leadership, research, or work in social justice and health disparities were associated with the interview invitation.	N = 188,500
15	Gray et al. (2023) [41]	Retrospective	Letters of recommendation (LOR) for pediatric surgical fellowship applications	To analyze the prevalence and type of bias in LORs using NLP.	Demographics were extracted from submitted applications. The Valence Aware Dictionary for Sentiment Reasoning (VADER) model was used to calculate polarity scores, and the National Research Council dataset was used for emotion and intensity analysis.	Differences in the intensity of emotions, particularly "anger," were statistically significant between racial and gender groups. While the types of emotions identified were highly similar, the intensity of these emotions revealed differences that may influence an individual's likelihood of successful matching.	N = 701
16	Guillen-Grima et al. (2023) [42]	Retrospective	Spanish Medical Residency Entrance Examination (MIR) questions	To assess the performance of the GPT-3.5 and GPT-4 language models in passing the MIR examination.	The LLMs were presented with 182 MIR examination questions in Spanish and English, and their performance was analyzed, including across different medical specialties, between theoretical and practical questions, and in terms of error proportions and severity.	GPT-4 outperformed GPT-3.5, scoring 86.81% in Spanish. While the models performed well, with error rates below 13.2%, understanding the severity of errors is critical when considering AI's potential role in real-world medical practice and its implications for patient safety.	
17	Booth et al. (2024) [43]	Retrospective	Anesthesiology graduate medical education narrative feedback	To fine-tune several large language models (LLMs) to explore the tradeoff between model complexity and performance while classifying narrative feedback on trainees into the ACGME sub-competencies.	The authors fine-tuned transformer-based LLMs (BERT-base, BERT-medium, BERT-small, BERT-mini, BERT-tiny, and SciBERT) to predict ACGME subcompetencies on a dataset of 10,218 feedback comments and compared their performance to a previous FastText model.	No models were superior to FastText, but BERT-mini performed comparably while being 94% smaller, which may allow for faster deployment and enhanced data privacy.	
18	Milutinovic et al. (2024) [44]	Retrospective	Cardiovascular medicine questions from the Medical Knowledge Self-Assessment Program (MKSAP)	To analyze the ability of ChatGPT to answer board-style cardiovascular medicine questions.	The performance of ChatGPT (versions 3.5 and 4), internal medicine residents, and internal medicine and cardiology attendings in answering 98 multiple-choice questions from the Cardiovascular Medicine Chapter of MKSAP was evaluated.	ChatGPT-4 demonstrated an accuracy of 74.5%, comparable to internal medicine residents and attendings but significantly lower than cardiology attendings (85.7%). Subcategory analysis revealed no statistical difference between ChatGPT and physicians, except in valvular heart disease and heart failure.	
19	Vasan et al. (2024) [45]	Retrospective	Otolaryngology residency application letters of recommendation (LORs)	To utilize natural language processing and machine learning (ML) models using LOR text to predict interview invitations for otolaryngology residency applicants.	LORs were retrospectively retrieved, preprocessed, and vectorized using three techniques (CountVectorizer, TF-IDF, and Word2Vec), and then trained and tested on five ML models (logistic regression, naive Bayes, decision tree, random forest, and support vector machine).	The two best-performing ML models in predicting interview invitations were the TF-IDF vectorized decision tree and CountVectorizer vectorized decision tree models, providing better-than-chance predictions.	N = 1642
20	Heath et al. (2024) [46]	Retrospective	Work-based assessments (WBAs) of standardized resident-patient encounters	To explore gender differences in WBA ratings as well as narrative comments when scripted performance was known.	Multivariable regression was used to assess gender differences in mean entrustment ratings, and natural language processing categories (masculine, feminine, agentic, and communal words) were used to determine associations of word use in the narrative comments with resident gender, race, and skill level, as well as faculty demographics.	Significant differences in entrustment ratings were found between women and men standardized residents, and feminine terms were more common in comments about what women residents did poorly, even after adjusting for the faculty's entrustment ratings.	N = 1527

The studies summarized in the table make valuable contributions to the growing body of research on the application of NLP and ML in medical education and graduate medical training. These computational approaches have demonstrated the ability to analyze diverse data sources, identify biases, automate assessment processes, and provide insights into the factors that influence trainee development and performance. As the field continues to evolve, the integration of these technologies into educational practices holds the promise of enhancing the assessment and support of medical trainees.

The papers reviewed in the provided table highlight the growing application of NLP and ML techniques in medical education and graduate medical training. These studies demonstrate the potential of these computational approaches to analyze various types of data, including residency applications, letters of recommendation, assessment narratives, and social media presence.

Several key findings emerge from the synthesis of results in the following domains.

Predicting Residency Performance and Matching

Studies by Ortiz et al. (2023), Drum et al. (2023), and Vasan et al. (2024) utilized NLP and ML models to analyze application materials, such as narrative letters of recommendation and experience entries, to predict interview invitation and match outcomes for residency applicants [38,39,45]. These models showed moderate to high predictive accuracy, suggesting the value of language-based features in assessing candidate potential.

Identifying Bias in Assessments

Researchers have employed NLP techniques to investigate potential gender and racial biases in residency evaluations and letters of recommendation. Studies by Sarraf et al. (2021), Gray et al. (2023), and Heath et al. (2024) found linguistic differences and variations in the intensity of emotions expressed in these materials, which may influence the perceived performance and success of trainees from underrepresented groups [31,41,46].

Automating Assessment Processes

NLP models have demonstrated the ability to classify the quality of feedback provided to trainees, as shown in the studies by Solano et al. (2021), Ötleş et al. (2021), and Spadafore et al. (2024). These findings suggest that automated systems could assist in the efficient review and improvement of assessment practices, potentially complementing human evaluations [29,30,33].

Understanding Entrustment and Competency Development

Researchers have used NLP to analyze narrative comments from workplace-based assessments and entrustable professional activity (EPA) evaluations, as reported by Stahl et al. (2021), Yilmaz et al. (2022), and Milutinovic et al. (2024) [28,36,44]. These studies provide insights into the factors that influence trainee autonomy and the development of competencies, informing educational interventions.

Evaluating AI Capabilities

The recent study by Guillen-Grima et al. (2023) explored the performance of LLMs, such as GPT-3.5 and GPT-4, in passing a Spanish medical residency entrance examination [42]. While the models performed well, the authors emphasize the importance of understanding the severity of errors when considering the potential role of AI in medical practice.

Discussion

The studies summarized in the table demonstrate the growing application of NLP and ML techniques in the realm of medical education and graduate medical training. These computational approaches have the potential to enhance various aspects of the assessment and selection processes for medical trainees.

One of the key areas where NLP and ML have shown promise is in predicting residency performance and match outcomes. Studies by Ortiz et al. (2023), Drum et al. (2023), and Vasan et al. (2024) utilized these techniques to analyze application materials, such as narrative letters of recommendation and experience entries, and achieve moderate to high predictive accuracy for interview invitations and match success [38,39,45]. This aligns with the findings of Mahtani et al. (2023), who employed NLP to analyze the relationship between applicant’s narrative experience entries and subsequent interview invitation, highlighting the potential of language-based features in assessing candidate potential [40].

The detection of bias in assessments is another area where NLP and ML have been applied. The studies by Sarraf et al. (2021), Gray et al. (2023), and Heath et al. (2024) found linguistic differences and variations in the intensity of emotions expressed in letters of recommendation and performance evaluations, which may influence the perceived success of trainees from underrepresented groups [31,41,46]. These findings align with the broader literature on the prevalence of bias in medical education, as reported by Dayal et al. (2017), Bludevich et al. (2021), and Mueller et al. (2020) [47-49]. The ability of NLP and ML to systematically identify these biases could inform efforts to improve the fairness and equity of assessment practices.

The potential for NLP and ML to automate and enhance assessment processes is also evident in the reviewed studies. Solano et al. (2021), Ötleş et al. (2021), and Spadafore et al. (2024) demonstrated the use of these techniques to classify the quality of feedback provided to trainees and analyze narrative comments from workplace-based assessments and EPA evaluations [29,30,33]. These findings are consistent with the work of Kalet et al. (2010), who explored the use of NLP to provide real-time feedback on clinical performance, and the research of Otleş et al. (2021), who reported an NLP-based system to analyze the quality of feedback in surgical training [33,50].

Furthermore, the studies by Yilmaz et al. (2022), Stahl et al. (2021), and Milutinovic et al. (2024) provide insights into the factors that influence trainee autonomy and the development of competencies, informing educational interventions [28,36,44]. This aligns with the broader efforts to understand and support the progression of medical trainees through the use of NLP and ML, as evidenced by the work of Grządzielewska et al. (2021) and Neves et al. (2021) [10,51].

The recent study by Guillen-Grima et al. (2023) on the performance of LLMs (GPT-3.5 and GPT-4) in passing a Spanish medical residency entrance examination highlights the importance of understanding the severity of errors when considering the potential role of AI in medical practice [42]. This concern is echoed in the broader literature by Cabitza et al. (2017), Knudsen et al. (2024), and Vora et al. (2023), where researchers have cautioned about the need for careful evaluation of the safety and reliability of AI systems in healthcare settings [52-54].

Limitations and future recommendations

While the studies reviewed in this systematic review demonstrate the potential of NLP and ML in medical education and graduate medical training, there are several limitations and areas for future research.

Generalizability

Most of the studies were conducted at single institutions or within specific medical specialties, which may limit the generalizability of the findings. Expanding the research to diverse educational settings and larger, multi-institutional datasets would help validate the robustness and broader applicability of the computational approaches.

Ethical Considerations

The detection of bias in assessments using NLP and ML raises important ethical concerns regarding the fairness, transparency, and accountability of these systems. Future research should explore the development of ethical frameworks and guidelines to ensure the responsible and equitable deployment of these technologies in medical education.

User Engagement and Collaboration

Effective integration of NLP and ML tools into educational practices requires close collaboration between researchers, educators, and trainees. Future studies should actively involve end-users in the design, development, and evaluation of these systems to ensure they meet the needs and address the concerns of the medical education community.

Longitudinal Impact

The reviewed studies primarily focused on short-term outcomes, such as interview invitations and match success. Longitudinal research is needed to evaluate the long-term impact of NLP and ML-based interventions on trainee performance, well-being, and career trajectories.

Validation and Benchmarking

Standardized datasets and evaluation protocols would help facilitate the comparison and benchmarking of different NLP and ML models, enabling the identification of best practices and the most effective approaches for specific educational tasks.

Interpretability and Explainability

As the complexity of NLP and ML models increases, it is essential to develop methods that enhance the interpretability and explainability of their decision-making processes. This would foster trust, transparency, and the ability to understand the underlying factors driving the models' outputs.

## Conclusions

The studies reviewed in this systematic analysis demonstrate the growing application of NLP and ML techniques in the field of medical education and graduate medical training. These computational approaches have shown promise in predicting residency performance and match outcomes, identifying biases in assessments, automating feedback processes, and providing insights into the factors that influence trainee development and competency progression.

The integration of NLP and ML into educational practices holds the potential to enhance the fairness, efficiency, and personalization of the assessment and support systems for medical trainees. However, the successful implementation of these technologies requires addressing key limitations, such as generalizability, ethical considerations, user engagement, longitudinal impact, validation, and interpretability. As the field continues to evolve, future research should focus on addressing these challenges and expanding the evidence base to ensure the responsible and effective integration of NLP and ML in medical education. Collaborative efforts between researchers, educators, and trainees will be crucial in shaping the future of computational tools that support the development and success of the next generation of medical professionals.
